# TSH Receptor Reduces Hemoglobin S Polymerization and Increases Deformability and Adhesion of Sickle Erythrocytes

**DOI:** 10.1155/2024/7924015

**Published:** 2024-04-02

**Authors:** Evelyn Mendonça-Reis, Camila Cristina Guimarães-Nobre, Lyzes Rosa Teixeira-Alves, Leandro Miranda-Alves, Clemilson Berto-Junior

**Affiliations:** ^1^Grupo de Pesquisa em Fisiologia Eritróide-GPFisEri, Universidade Federal do Rio de Janeiro, Campus Macaé, Rio de Janeiro, Brazil; ^2^Programa de Pós-Graduação em Endocrinologia, Faculdade de Medicina, Universidade Federal do Rio de Janeiro, Rio de Janeiro, Brazil; ^3^Laboratório de Endocrinologia Experimental-LEEx, Instituto de Ciências Biomédicas, Universidade Federal do Rio de Janeiro, Rio de Janeiro, Brazil; ^4^Programa de Pós-Graduação em Farmacologia e Química Medicinal, Instituto de Ciências Biomédicas, Universidade Federal do Rio de Janeiro, Rio de Janeiro, Brazil

## Abstract

SCD is a hereditary disorder caused by genetic mutation in the beta-globin gene, resulting in abnormal hemoglobin, HbS that forms sickle-shaped erythrocytes under hypoxia. Patients with SCD have endocrine disorders and it was described that 7% of these patients have clinical hypothyroidism. Recent studies have shown that mature erythrocytes possess TSH receptors. Thus, we aimed to assess the effects of TSH on SCD erythrocytes. The experiments were conducted using different concentrations of TSH (1, 2, 3, and 5 mIU/L). In HbS polymerization assay, erythrocytes were exposed to TSH in hypoxia to induce polymerization, and measurements were taken for 30 minutes. The deformability assay was made using Sephacryl-S 500 columns to separate deformable from nondeformable cells. Static adhesion test utilized thrombospondin to assess erythrocyte adhesion in the presence of TSH. TSH at all contractions were able to reduce polymerization of HbS and increase deformability. The static adhesion of erythrocytes at the lowest concentrations of 1 and 2 mIU/L were increased, but at higher contractions of 3 and 5 mIU/L, static adhesion was not modulated. The results suggest that TSH has potential involvement in the pathophysiology of sickle cell disease by inhibiting HbS polymerization, positively modulating deformability and impacting static adhesion to thrombospondin.

## 1. Introduction

Thyroid stimulating hormone (TSH) is a glycoprotein hormone produced by the anterior pituitary, widely known for its role in the production of the hormones triiodothyronine (T3) and thyroxine (T4) by the thyroid gland [1]. TSH acts by binding to the TSH receptor (TSHR), which is a G protein-coupled receptor (GPCR) known to be present on the basolateral surface of thyroid follicular cells [2]. However, the TSH receptor has already been identified in tissues other than the thyroid, such as the anterior pituitary; hypothalamus; [3–5] ovary; testicle [6, 7]; skin [8, 9]; kidney [10, 11]; immune system; bone marrow; white and brown adipose tissue [12, 13]; orbital preadipocyte fibroblasts [14]; bones; NK cells [13, 15]; and also in erythrocytes [16].

Erythrocytes are anucleated, biconcave disk-shaped cells present in the blood. They are produced in the bone marrow, have a life of approximately 120 days and are composed of hemoglobin molecules (Hb), whose main function is to transport oxygen and carbon dioxide to all tissues of the body. Hb is a tetramer consisting of two subunits of globin chains, each linked to a heme group [17, 18]. Hemoglobinopathies are a group of diseases characterized by mutation and disfunction of hemoglobin. There are several hemoglobinopathies that can affect erythrocytes and their structures, such as sickle cell disease (SCD), which is an autosomal recessive genetic disease that affects approximately millions of people around the world, and in Brazil, each year, 3,500 children are born with SCD and 200,000 with sickle cell trait [19, 20].

When sickle erythrocytes are exposed to HbS deoxygenation in tissues with high oxygen demand, exposure of hydrophobic sites on HbS tetramers occurs, so the *β*1 and *β*2 chains bind to two hemoglobin molecules to hide the hydrophobic sites, initiating the formation of a polymer of HbS. These HbS polymers grow rapidly and form long fibers that increase cellular rigidity and distort the erythrocyte membrane, leading to sickling of erythrocytes, cellular energy failure and stress, dehydration, low deformability, impaired rheology, and premature hemolysis [20, 21].

SCD has several clinical manifestations, such as vaso-occlusion, which promotes ischemia-reperfusion injury, and is the predominant pathophysiology responsible for vaso-occlusive crisis (VOC), an acute systemic painful crisis [22]. Vaso-occlusion causes interaction between impaired blood rheology, increased adhesion of erythrocytes to inflammatory cells and vascular endothelium, and hemostatic activation [23]. The damage that sickling causes to erythrocyte membranes also promotes the exposure of adhesion molecules and binding sites that are not normally expressed, such as phosphatidylserine (PS), basal cell adhesion molecule-1/lutheran (B-CAM-1)./Lu), integrin-associated protein (IAP) and intercellular adhesion molecule-4 (ICAM-4) [20], and increased circulating thrombospondin-1, which is supposedly generated by activated platelets and increases the adhesion of erythrocytes to the endothelium through the CD47 receptor. In addition, thrombospodin-1 has already been described as an inhibitor of the vasodilatory, antiadhesive, and homeostatic effects of nitric oxide signaling pathways and also of vascular endothelial growth factor, which may affect the regulation of tissue perfusion and vascular tone, leading to inflammation frame [24, 25]. Another recurring problem in the pathophysiology of SCD is hemolytic anemia, which is influenced by the polymerization of HbS. It is already known that as the aging of patients with SCD, the risks of vasculopathies increase, characterized by systemic and pulmonary hypertension, endothelial dysfunction, and changes in the intima and smooth muscle of blood vessels. In addition to progressive vasculopathy, hemolysis causes anemia, fatigue, and cholelithiasis [26–28].

It has already been described that patients with SCD have metabolic and endocrine disorders, which may be the result of hypoxia, chronic anemia, iron overload, or genetic influence. [29] describes that 7% of patients with SCD have clinical hypothyroidism and high concentrations of TSH (6.4 mIU/L) [29]. These endocrine disorders can cause growth retardation and delayed puberty. Repeated blood transfusions and hemolysis can cause iron overload and consequent disruption of tissue vitalization during vaso-occlusive crisis and increase in inflammatory mediators that mainly cause metabolic and endocrine dysfunction [29–31]. [30] observes impaired thyroid microcirculation and decreased thyroid volume among patients with SCD, and these factors were related to disease duration, but the results were not related to thyroid function, suggesting that these disorders can happen independently of the accumulation of iron [30].

Thus, our work aims to study the effects of TSH, in a dose-dependent manner, on the erythrocytes of sickle cell patients, based on the parameters of polymerization, deformability, and static adhesion.

## 2. Methodology

### 2.1. Samples

The collection of sickle cell patient's blood (exclusively SS genotype) was performed at the Institute of Hematology Arthur de Siqueira Cavalcanti (HEMORIO) and was carried out after project approval by the Ethics Committee of the Federal University of Rio de Janeiro (Protocol 1032.889.952). The project is registered on Brazil platform under CAAE number 88140418.5.0000.5699 and was in conformity with standards set by the Declaration of Helsinki. Both men and women, over 16 years of age (for those being 16 or 17, with written consent given by the responsible person) were informed about the study and those who agreed to participate filled out the free informed consent form for collection of blood sample and subsequent use. Those taking controlled medication or having any other hemoglobinopathies (heterozygous) were excluded. Blood samples were collected in anticoagulant tubes with EDTA by puncture on antecubital fossa. The collected blood underwent a washing process (three times with PBS) to remove the plasma and buffy coat, leaving only the red blood cell concentrate (RBC), which were used in the experiments. All experiments were performed in triplicate and the final TSH concentrations used was 1, 2, 3, and 5 mIU/L.

### 2.2. HbS Polymerization Assay

For the HbS Polymerization assay, blood was collected from sickle cell patient in EDTA tubes and centrifuged for 5 minutes at 750 g. After removing plasma and the buffy coat, RBCs were washed three times with PBS under the same centrifugation conditions. In a 96-well plate, TSH was added at four different concentrations (1, 2, 3, and 5 mIU/L, final concentrations), along with 2% sodium metabisulfite (a HbS polymerization inducer that reacts with water-dissolved oxygen). The final RBC volume corresponded to a 1% hematocrit. After adding all reagents, the plate was immediately read at 700 nm absorbance for 30 minutes using a microplate reader (Tecan GENios), with readings taken at approximately 1-minute interval. The evaluation of HbS polymerization was based on the turbidity, resulting from the polymerization induced by metabisulfite, a deoxygenating agent. Control wells were also prepared, containing only erythrocytes and 2% sodium metabisulfite. Following the generation of the polymerization curve, statistical analysis was conducted based on the calculated area under the curve.

### 2.3. Static Adhesion

The experiment began by adding 50 *μ*L of thrombospondin in a PBS solution at a concentration of 2 *μ*g/mL to each well of a 96-well plate. Subsequently, the plate was incubated in a water bath for two hours. After this incubation period, thrombospondin was removed, and a 0.3% albumin solution in PBS was applied to block areas where thrombospondin did not bind, preventing nonspecific binding of RBCs. The plate was then returned to the water bath for an additional 30 minutes. Following the removal of the previously added medium, blood and the substances under study were introduced into RPMI medium (TSH at concentrations of 1, 2, 3, and 5 mIU/L) and incubated for an additional 2 hours. Carefully, the red blood cells that did not adhere were removed and washed three times with PBS. Finally, 100 *μ*L of MilliQ water was added to lyse all adhered erythrocytes (resulting in a final volume of 100 *μ*L), and spectrometric quantification of hemoglobin was performed by reading at 540 nm in a microplate reader.

### 2.4. Deformability

To conduct the deformability experiment, we added 125 *μ*L of Sephacryl-S 500 (a highly versatile gel filtration resin) to the Eppendorfs, washed the columns three times using PBS, and centrifuged them for 5 minutes at 1000 g. Subsequently, we prepared oxygenated controls (PBS) and deoxygenated controls (PBS with 2% of the final volume of sodium metabisulfite). The substances (TSH 1, 2, 3, and 5 mIU/L in PBS with sodium metabisulfite at 2%) were then prepared, and red blood cells (1% hematocrit) were added and incubated for 10 minutes. The content of interest was added to the previously prepared Sephacryl columns and then taken and centrifuged for 5 minutes at 3000 g to separate sickled cells from intact cells, thus distinguishing between deformable (cells in the bottom of the column) and nondeformable cells (those on top of the column). Subsequently, the supernatant (nondeformable cells) was removed and added to another tube with 1 mL of MilliQ water. In the Eppendorfs with precipitate (deformable cells), 1 mL of MilliQ water was also added (adding water is important to lyse erythrocytes for hemoglobin quantification at 540 nm and 700 nm). Again, the tubes were centrifuged for 5 minutes at 1000 g to separate the Sephacryl resin from the samples, and the lysate contents dissolved in water. To perform the reading, 200 *μ*L of the supernatant was placed in a 96-well plate, and the reading was performed using optical density (OD) at 540 nm and 700 nm for deformable and non-deformable phases of the RBCs, utilizing a Tecan Genius microplate reader. The deformability result for red blood cells, under the analyzed conditions, is obtained by calculating the absorbance of deformable and nondeformable hemoglobin. To do this, a calculation is performed with the result of the absorbance of the deformable and nondeformable parts, as described: [OD (542 nm–700 nm) of Hb in the deformable fraction of red blood cells/(OD (542 nm–700 nm) of Hb in deformable red blood cells + fraction of nondeformable red blood cells)] *∗* 100 [32].

### 2.5. Statistical Analysis

GraphPad Prism 9 was used to plot the graphs and to perform statistical analysis, with all data expressed as Mean ± SEM. An ANOVA with Tukey's posttest was used for analysis of the significant differences (^*∗*^ for *p* < 0.05, ^*∗∗*^ for *p* < 0.01 and ^*∗∗∗*^ for *p* < 0.001) with a confidence interval of 95%. For hemoglobin S polymerization curves, a nonlinear fit line calculus with a fourth order polynomial equation calculated with 95% confidence intervals.

## 3. Results

**Figure 1:**
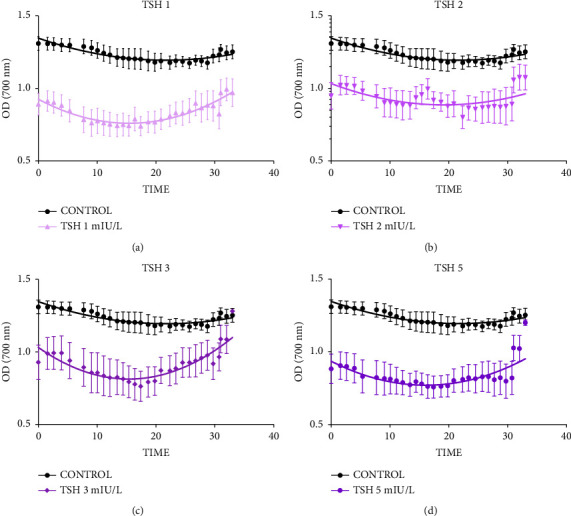
TSH promotes inhibition of HbS polymerization. Reading performed every one minute (approximately) for 30 minutes. The rate of polymerization with TSH 1 mIU/L (a), TSH 2 mIU/L (b), TSH 3 mIU/L, and (c) e TSH 5 mIU/L (d). Reading taken at 700 nm absorbance. *N* = 4. The results expressed as the mean ± SEM, with 95% confidence interval.

**Figure 2:**
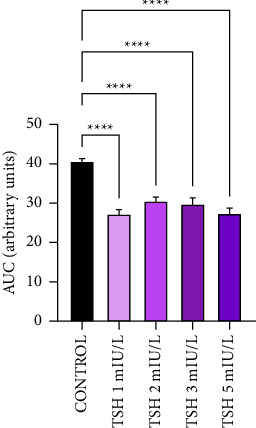
TSH inhibits *ex vivo* HbS polymerization. The area under the curve (AUC) plot of polymerization inhibition with TSH at concentrations of 1, 2, 3, and 5 mIU/L and control. Reading taken at 700 nm absorbance. *N* = 4. The results expressed as the mean ± SEM. ^*∗∗∗∗*^*p* < 0.0001 vs control, with 95% confidence interval in one-way ANOVA and Tukey posttest.

From the graphs of polymerization measurement, it was observed that in the presence of TSH there was a decrease in polymerization rate in all its concentrations (1, 2, 3, and 5 mIU/L), with the control starting at 1.3 of optical density, while after the addition of TSH at all concentrations, polymerization started at approximately 0.9 optical density (Figure 1). Quantification of the area under the curve showed that the decrease was statistically significant in all concentrations of TSH, which were 33.01; 24.83; 26.71, and 32.52%, respectively, for concentrations of 1, 2, 3, and 5 mIU/L (Figure 2).

**Figure 3:**
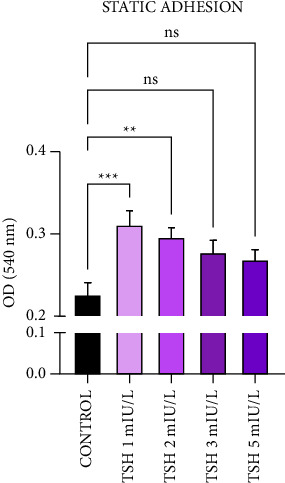
TSH increases adherence of sickle erythrocytes to thrombospondin at lower concentrations (1 and 2 mIU/L) and does not change at higher concentrations (3 and 5 mIU/L). The static adhesion experiment with thrombospondin was performed with TSH concentrations 1, 2, 3, and 5 mIU/L, and control. Reading taken at 540 nm absorbance. *N* = 4. Results expressed as mean ± SEM. ^*∗∗*^*p* < 0.01, ^*∗∗∗*^*p* < 0.001 vs control, with 95% confidence interval in one-way ANOVA and Tukey posttest.

For the static adhesion experiment (Figure 3), increase was significant at the lowest concentrations of TSH, 1 and 2 mIU/L, and this increase, compared to the control, was 37.65 and 31.10%, respectively. At the highest concentrations, 3 and 5 mIU/L of TSH, the increase compared to the control was 22.56 and 18.58%, respectively, although it was not statistically significant.

**Figure 4:**
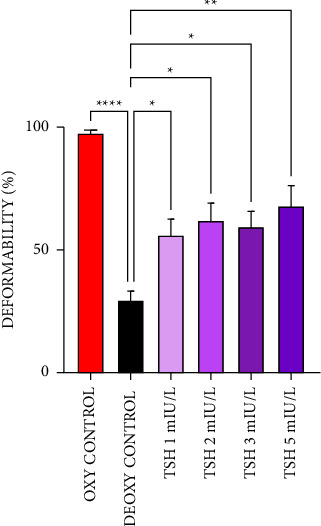
TSH was able to increase the deformability of sickled erythrocytes. In this experiment, concentrations of 1, 2, 3, and 5 mIU/L of TSH were used, two controls were also made, one oxygenated and the other deoxygenated (with metabisulphite). The samples were read at absorbances of 540 and 700 nm to calculate the values used in the graph. *N* = 3. Results expressed as mean ± SEM. ^*∗*^*p* < 0.05, ^*∗∗*^*p* < 0.01, and ^*∗∗∗∗*^*p* < 0.0001 vs control, with 95% confidence interval in one-way ANOVA and Tukey post-test.

In deformability test, an oxygenated control was used, which is possible to observe almost 100% deformability. By inducing HbS polymerization, the deoxygenated control has only 29.56% of deformability, a decrease of 70.44%, which was significant compared to control. With the addition of TSH concentrations, there was a significant increase in erythrocyte deformability when compared to the deoxygenated control, of 26.43; 32.47; 29.85, and 38.33%, respectively, for concentrations of 1, 2, 3, and 5 mIU/L of TSH (Figure 4).

## 4. Discussion

The erythrocyte has already been observed as more than just an oxygen carrier and that it has several receptors, which when activated play different roles in controlling the cell physiology. The TSH receptor, for example, has already been described on the erythrocyte [33] and in 2020, Evelyn et al. observed different activated pathways in a hypoosmotic stress situation and showed that TSH can improve the resistance of red blood cells to hemolysis and that this effect was through inhibition of the AMPK-dependent pathway and concomitant activation of the PI3K/Akt signaling pathway [34]. In the current work, we aimed to observe important parameters of sickle erythrocyte, such as HbS polymerization, adhesion to extracellular matrix proteins and deformability when exposed to TSH at concentrations of 1, 2, 3, and 5 mIU/L.

Polymerization can be considered the primary event of sickle cell disease, since when HbS is in a state of hypoxia or deoxygenation, polymerization occurs, causing a change in the conformation of erythrocytes and leading to other factors of the pathophysiology of the disease, such as increased adherence and decreased deformability [20, 35, 36].

The polymerization results showed that TSH was able to decrease polymerization compared to the control by an average of 30%, knowing that this is a key point of sickle cell disease, this decrease can be crucial for patients with sickle cell disease.

It has already been observed that one of the mechanisms involved in polymerization is cell dehydration, which can occur via the Gardos channel [37]. The Gardos channel is normally activated so that rupture of the erythrocyte is prevented, occurring an increase in the output of potassium and chloride ions from the cell, resulting in a loss of water and a decrease in cell volume. It has already been suggested that prostaglandin E2 (PGE2), endothelin (ET-1), and cytokines have the potential to activate the Gardos channel in healthy and sickled erythrocytes [35, 38, 39]. Thus, we believe that TSH may be acting via the Gardos channel to reduce the effects of HbS polymerization, by inhibiting this channel activity. Although, more experiments need to be performed in order to validate this hypothesis.

When assaying the static adhesion of erythrocytes to thrombospondin, we observed that the lowest concentrations of TSH (1 and 2 mIU/L) significantly increased adhesion when compared to control, but the highest concentrations of TSH (3 and 5 mIU/L) did not have a significant increase. This has already been observed that sickle cell patients have functional hypothyroidism and TSH levels are approximately 6.4 mIU/L in one study [29]. Therefore, as 3 and 5 mIU/L concentrations were not statistically significant, we believe that in sickle cell patients, high levels of TSH do not affect adherence to the vascular endothelium regarding thrombospondin binding. For a broader observation in relation to adherence to the vascular endothelium to other adhesion molecules, further experiments will be needed.

When we verified the modulation of TSH in the deformability of sickle erythrocytes, we observed a dose-dependent increase in deformability at all TSH concentrations (1, 2, 3, and 5 mIU/L). Deformability is crucial for the erythrocytes to reach the capillaries that perfuse the organs and then be able to perform gas exchange, which is why this result is significant [20]. Furthermore, sickle RBC have been observed to be less deformable than healthy RBC, meaning they exhibit significantly higher membrane shear modulus and viscosity. This phenomenon may occur due to fatigue caused by cyclic hypoxia, a characteristic feature of SCD [40, 41]. The decrease in deformability is due to the polymerization of hemoglobin S, which forms axial bundles due to the interaction of valine-6 with hydrophobic residues of leucine and phenylalanine, in addition, the concomitant dehydration that occurs due to the loss of water through the Gardos channel collaborates so that the erythrocytes assume the rigid sickle shape characteristic of sickle cell anemia [20, 36, 37, 39]. Just as we believe that TSH may be acting via the Gardos channel to improve polymerization, it may also be acting to improve deformability. In this sense, inhibition of the channel may keep a high-water content within the cell improving the deformability, despite the low oxygen tension.

## 5. Conclusion

**Figure 5:**
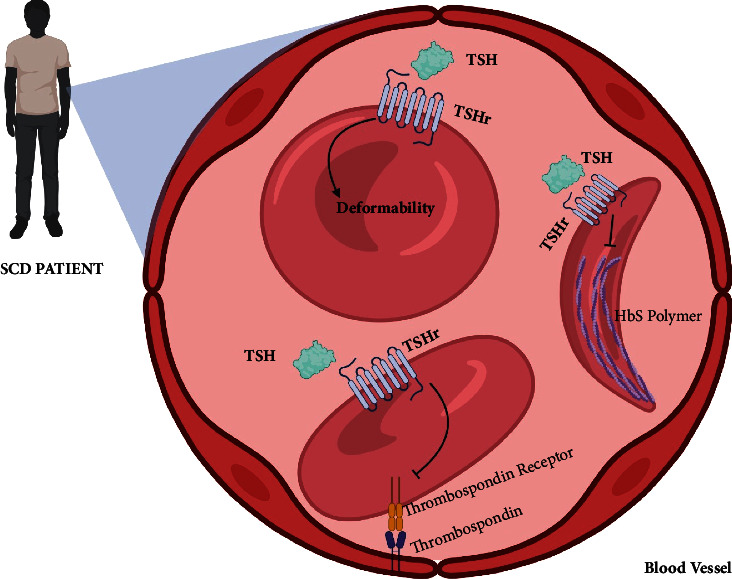
Scheme showing the importance of TSH in HbS polymerization, adhesion of erythrocyte to thrombospondin, and deformability of erythrocytes from patients with sickle cell disease.

Based on the results obtained in this study, we can conclude that TSH has potential participation in sickle cell disease pathophysiology (Figure 5). The findings suggest that TSH can mitigate HbS polymerization and deformability, which are critical events of sickle cell disease. And although the results of static adhesion with thrombospondin were not significant at the highest concentrations of TSH (3 and 5 mIU/L) used in this study. The result was of relevance since the TSH concentrations are comparable to those observed in patients with sickle cell disease. It is clear the importance of constant observation of TSH levels in sickle cell patients, and that there should also be tests to observe the thyroid morphology in search of alterations. Overall, more studies are needed to elucidate the precise molecular mechanisms underlying these effects and to evaluate the therapeutic potential of TSH in the management of sickle cell disease.

## Data Availability

The primary research data that support the findings of this study can be provided under request to correspondent author.
